# Effectiveness of the Hypertension Screening Corner in Enhancing the Cascade of Care at Primary Healthcare Center Level: Evidence from Zambezia, Mozambique

**DOI:** 10.5334/gh.1339

**Published:** 2024-07-10

**Authors:** Anna Sartorello, Roberto Benoni, Lucy Ramirez, Aldo Mundjane, Frederic Kalombola, Alfredo Ramos, Edgar Meque, Paolo Massaro, Neusa Jessen, Giovanni Putoto, Albertino Damasceno

**Affiliations:** 1 Department of Diagnostic and Public Health, University of Verona, Verona, Italy; 2 Doctors with Africa CUAMM, Maputo, Mozambique; 3 Department of research, training and health surveys, National Institute of Health, Maputo, Mozambique; 4 Sofala Provincial Health Service, Ministry of Health, Beira, Mozambique; 5 Operational Research Unit, Doctors with Africa CUAMM, Padova, Italy; 6 Faculty of Medicine, Eduardo Mondlane University, Maputo, Mozambique

**Keywords:** Hypertension, Cascade of care, Screening, Cardiovascular diseases, Mozambique, Primary healthcare

## Abstract

**Background:**

Hypertension is the leading cause of cardiovascular disease, whose death burden is dramatically increasing in sub-Saharan Africa. To curb its effects, early diagnosis and effective follow-up are essential. Therefore, this study aims to evaluate the impact of a hypertension screening corner on the hypertension care cascade at the primary healthcare level.

**Methods:**

A prospective cohort study was conducted between October 2022 and March 2023 in two PHCCs in Zambezia (Mozambique). The study involved a demographic and socioeconomic status (SES) questionnaire for those screened. Patients with blood pressure (BP) > 140/90 mmHg were given a follow-up questionnaire regarding the care cascade. The four cascade steps were: medical visit, diagnosis confirmation, follow-up visit, and recalling the follow-up appointment. The odds ratio (OR) of reaching each step of the cascade was assessed by binomial logistic regression.

**Results:**

Patients with BP > 140/90 mmHg were 454, and 370 (86.0%) completed both study phases. Individuals attending the medical visit were 225 (60.8%). Those with low SES had a higher probability of visit attendance than those with middle (OR = 0.46, 0.95CI[0.23–0.88] p = 0.020) and high (OR = 0.21 0.95CI[0.10–0.42], p < 0.001). Hypertension diagnosis was confirmed in 181 (80.4%), with higher probability in the low SES group compared to the middle (OR = 0.24 IC95[0.08–0.66], p = 0.007) and high (OR = 0.23, IC95[0.07–0.74], p = 0.016) groups. The OR to complete step 1 and step 2 were higher for older age groups. A follow-up appointment was received and recalled by 166 (91.7%) and 162 (97.6%) patients, respectively.

**Conclusions:**

The hypertension corner proved to be a useful tool for effective screening of hypertension with satisfactory retention in care, especially for people with lower socio-economic status.

## Introduction

Noncommunicable diseases (NCDs) are responsible for 41 million deaths every year, accounting for 74% of all deaths globally. Of these, 77% occur in low- and middle-income countries (LMIC) [1]. Cardiovascular disease (CVD) is the leading cause of death among NCDs, and hypertension is one of its major causes [2]. According to data from the Global Burden of Disease 2019, between 1990 and 2019, disability-adjusted life years (DALYs) due to high systolic blood pressure decreased by 34% in high-income countries, while it increased by 24% in low- and middle-income countries (LMICs) [3].

The burden of NCDs in the African region is mainly due to the epidemiological transition linked to globalization and lifestyle changes, with an increased prevalence of risk factors such as unhealthy diets, insufficient exercise, dyslipidemia, and obesity [4]. Moreover, other risk factors such as pollution and urbanization are emerging [4]. In addition, systemic factors such as the lack of services and human and material resources, as well as socio-economic factors, make sub-Saharan Africa one of the areas with the lowest rate of access to care and consequently with a higher risk of CVD death [5].

The prevalence of hypertension is rising globally, and the WHO reports a prevalence of hypertension of 27% in the African region [6]. In Mozambique, despite the population being very young (63.3% under 24 years) [7], a prevalence of hypertension between 14.1% and 25.2% has been reported [8,9]. Although older age is a known risk factor for hypertension, it was found that in Mozambique, blood pressure measurement and diagnosis is more common among younger age groups (36.2% and 13.6% respectively in those aged 20–30 years) than older ones (23.3% and 5.8% in those over 60 years) [9].

There are numerous studies on the prevalence and determinants of hypertension in sub-Saharan Africa (SSA), but information on hypertension awareness, treatment, and control is limited [10,11]. Low awareness contributes to low treatment and control rates, influenced by a poor network of public health facilities, poor distribution of essential drugs, and poor access to care [12]. Key actions in the response to NCDs include primary prevention, early diagnosis, and appropriate treatment. Despite an increase in the NCD visits in LMICs, access to healthcare for chronic disease remains low with primary healthcare centers (PHCC) playing a pivotal role in disease control and prevention [13]. Screening campaigns can help increase awareness of the risks associated with hypertension and the benefits of early diagnosis and management [14].

In Mozambique, a new approach was tested introducing the *hypertension corner* (HC), a suitably equipped space at the entrance to the PHCCs. At the HC a trained community healthcare worker invites and measures blood pressure (BP) to all adults consenting to do so, using an automatic BP machine. If the BP is higher than 140/90 mmHg in two subsequent measurements, the person is referred to the appropriate outpatient clinic. A pilot study reported a significant increase in the number of new hypertensive patients diagnosed by the health center in 6 months after the introduction of the HC [15].

The cascade of care, or continuum of care, is a model used to assess patient retention through the sequential steps of care required to achieve a positive outcome. The cascade is used to identify areas where there are gaps in care delivery and to implement improvement interventions. It can be used to assess the effectiveness of a population screening intervention. In LMICs, it has been suggested that the care cascade should also include the screened population, in addition to those connected to quality care and those adhering to prescribed treatment, allowing for a better measurement of the coverage and performance of health systems [16].

Therefore, the primary objective of this study is to evaluate how the screening strategy implemented in Zambezia, Mozambique, through the hypertension corner, influences the cascade of care for patients with high blood pressure. Secondary objectives are the description of the beneficiaries of the hypertension corner and the evaluation of sociodemographic and socioeconomic factors that may influence each step of the cascade of care.

## Methods

### Study design

A prospective cohort study was conducted to assess the impact of the *hypertension corner* on the hypertension cascade of care from October 2022 to March 2023 in Quelimane, province of Zambezia, Mozambique.

### Population and setting

The study was conducted in two PHCCs in the city of Quelimane, namely the “17 de Setembro” PHCC and the “Coalane” PHCC, where the hypertension corner was active.

Mozambique is a country in south-eastern Africa and is divided into ten provinces. Quelimane is the capital city of Zambezia, a region in the north of Mozambique. It has an estimated population of 350,000 inhabitants, 36,7% of whom are over 25 years of age [17].

Individuals aged 25 years or older who accessed the PHCC were considered eligible for this study. Before being enrolled in the cohort, they were asked by a community health worker (CHW) located in the hypertension corner to sign an informed consent to participate. The exclusion criteria were declining to participate, not being able to give informed consent, and already being treated for chronic diseases (i.e., diabetes, cardiovascular diseases, chronic respiratory diseases, thyroid diseases, and neoplastic diseases).

### Data collection

The study recruitment took place in the HC located at the PHCC and consisted of two phases. First, people underwent blood pressure measurements by a trained CHW on a voluntary basis and, if their blood pressure was equal or above 140/90 mmHg after the second measurement, they were asked to participate in the study; if they agreed, they signed the informed consent and were given a questionnaire. The following information was collected: socio-demographic characteristics, household and individual socioeconomic factors, and knowledge and habits regarding hypertension **(Annex 1)**. Subsequently, all patients with high BP were referred to the chronic diseases outpatient clinic to confirm hypertension. Phase two of the study consisted of a second questionnaire on the management and treatment of hypertension. The second questionnaire **(Annex 2)** was either filled out at the health unit after the medical examination or by telephone two weeks after the positive screening by the same trained CHW.

### Steps of the Cascade of Care

The treatment cascade for hypertension consisted of five steps:

- **Stage 0: Positive screening**. After PHCC access and hypertension screening, subjects who tested positive for hypertension screening were referred for specialist consultation.
- **Stage 1: Underwent the medical visit**. Patients could go to the specialist outpatient clinic on the same day as the screening or within the following 15 days.
- **Step 2: Confirmed diagnosis**. If the diagnosis was confirmed after a third BP measure, the doctor explained the hypertension disease to the patient and prescribed the necessary treatment.
- **Stage 3: Received a follow-up appointment**. The patient with hypertension received an appointment for a follow-up visit to monitor symptoms and check the treatment.
- **Stage 4: Recalled the follow-up appointment**. The patient can recall the follow-up appointment date.

### Variable definition

Hypertension was defined as a blood pressure measurement equal to or higher than 140 mmHg for systolic and 90 mmHg for diastolic in two 5-minute repeated measurements. The CHW was trained accordingly to the WHO guidelines using an automated BP machine with an adequate cuff size [18].

Socioeconomic status (SES) was measured based on the available literature (papers from research conducted in LMICs dealing with the creation of an SES index were included), considering household and individual factors, such as education and employment [19,20]. Education and employment were assessed according to International Standard Classifications [21,22]. The most common jobs in Mozambique have been included and ordered according to the relative rank and income of each occupation [19]. The SES index was built using principal component analyses (PCA) of the abovementioned variables, and then categorized into wealth tertile (low, medium, high) [23].

Basic knowledge of hypertension and its risk factors was assessed by asking each individual what the most common symptoms of hypertension were (i.e., headaches and dizziness) and how important a low-salt diet was. The index ranged from 0 to 4.

### Statistical analysis

The descriptive analysis of the sample used frequencies and proportions for qualitative variables; means and standard deviation, or medians and quartiles for quantitative variables.

Differences in sample distribution were tested with the t-test or Mann-Whitney test for continuous variables and the χ^2^ or Fisher’s exact test for categorical variables, as appropriate.

The multivariable association between individual characteristics and the steps of the cascade of care was explored using binomial logistic regression, including all variables. The response variables were medical consultation (yes/no), confirmed diagnosis (yes/no), follow-up appointment (yes/no), and recalling the appointment (yes/no). Independent variables were age, sex, and SES index categories. Results were presented as odds ratio (OR) with 0.95 confidential interval (CI). Post-hoc pairwise comparison between SES index and age groups in the logistic regressions was carried out through Tukey’s test.

A p-value < 0.05 was considered significant. All analyses were performed using the R software (version 4.1.1).

### Ethics

The inclusion of a patient in this protocol did not require any additional exams or invasive medical procedure besides those normally needed for clinical routine. The research was performed following the ethical standards of the 1964 Declaration of Helsinki and was approved by the Institutional Bioethics Committee for Health (Comité Institucional de Bioética para a Saúde, CIBS) – Zambezia, on September 9, 2022 (protocol number 97/CIBS-Z/22).

## Results

### Sample characteristics

**Figure 1:**
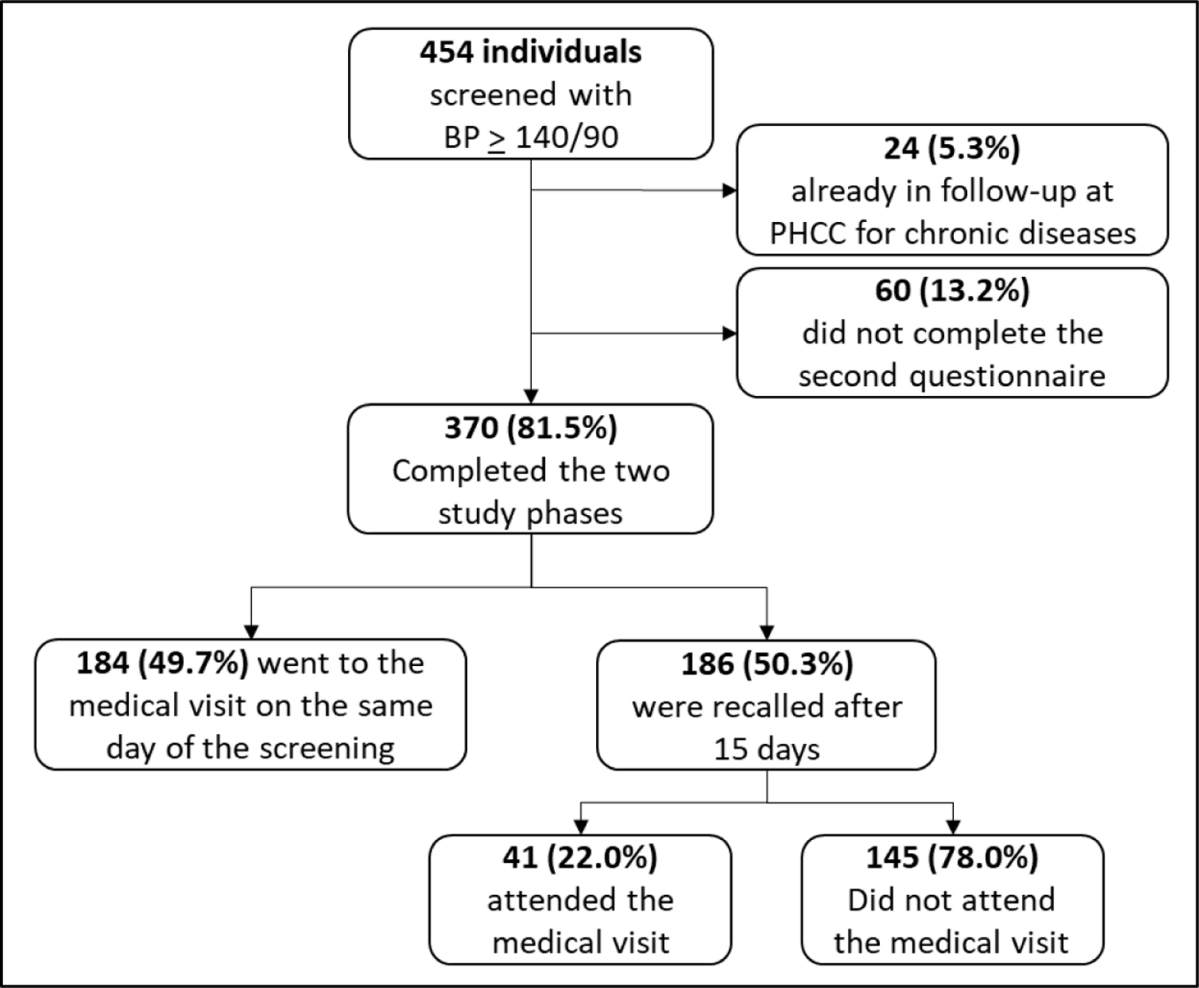
Flowchart of study inclusion criteria. BP = Blood pressure, PHCC = Primary Health Care Center.

In the study period, 6,659 individuals were BP screened and 454 (6.8%) presented a blood pressure >140/90 mmHg. After applying the inclusion/exclusion criteria, 430 individuals were included in the study (Figure 1).

Of these, 275 (64.0%) were female. The descriptive data of the sample are summarized in Table 1.

**Table 1:** Number and frequencies of the socioeconomic status (SES) index group and variable included in SES index estimation distinguished by sex.


	FEMALES	MALES	OVERALL

	(n = 275)	(n = 154)	(n = 430)

**SES index (NA = 81)**			

Low	107 (38.9)	16 (10.4)	123 (29.6)

Medium	79 (28.7)	27 (17.5)	106 (28.6)

High	50 (18.2)	70 (45.4)	120 (27.9)

**Education (NA = 1)**			

Illiterate	112 (40.7)	22 (14.3)	134 (31.2)

Primary school	67 (24.4)	39 (25.3)	107 (24.9)

Middle school	54 (19.6)	39 (25.3)	93 (21.6)

High school	29 (10.5)	36 (23.4)	65 (15.1)

University or higher	13 (4.7)	18 (11.7)	31 (7.2)

**Employment (NA = 35)**			

Subsistence agriculture	22 (8.0)	22 (17.5)	203 (47.2)

Self-occupied	175 (63.6)	27 (26.0)	65 (15.1)

Formal paid work	25 (9.1)	40 (15.6)	80 (18.6)

Unemployed	30 (10.9)	50 (12.3)	44 (10.2)

**Household variables**			

Non-perishable ceiling (NA = 2): yes	233 (84.7)	144 (93.5)	377 (87.7)

Washable floor (NA = 3): yes	133 (48.4)	90 (58.4)	223 (51.9)

Telephone (NA = 1): yes	176 (64.0)	137 (89.0)	313 (72.8)

Television (NA = 2): yes	170 (61.8)	113 (73.4)	283 (65.8)

Electricity (NA = 9): yes	244 (88.7)	138 (89.6)	373 (86.7)

Running water (NA = 1): yes	190 (69.1)	129 (83.8)	319 (74.2)

**Toilet** (NA = 2)			

With a washable floor	68 (24.7)	27 (17.5)	95 (22.1)

With perishable ceiling	191 (69.5)	124 (80.5)	315 (73.2)

None	15 (5.5)	2 (1.3)	17 (3.9)

**Means of transpor**t (NA = 16)			

None	188 (68.4)	68 (44.2)	256 (59.5)

Motorbike	42 (15.3)	50 (32.5)	92 (21.4)

Moto	44 (16.0)	39 (25.3)	83 (19.3)

Car	8 (2.9)	9 (5.8)	17 (3.9)


Females had a significantly higher prevalence of SES index low, (47.2% vs. 15.2%). Indeed, women were more likely to be illiterate (40.7% vs. 14.3%) and had a higher prevalence of subsistence farming as the main household income (63.3% vs. 26.0%).

The mean knowledge index score was 2.02 (SD = 0.77), i.e., most individuals considered a low-salt diet to be relevant (n = 365, 84.9%). However, few have been able to correctly identify at least one symptom of hypertension (n = 81, 18.8%). In the study sample, knowledge of hypertension differed significantly according to SES index, being higher in the high group than in both the low and middle groups (p < 0.001).

There were no differences according to sex (p = 0.627) and age (p = 0.175).

Of the 430 people who tested positive for hypertension, 329 (76.5%) were asymptomatic, 72 (52.5%) had already taken antihypertensive drugs in the past, 137 (31.8%) were aware of their condition, and 150 (34.9%) had never had their blood pressure measured. There were no significant differences between those who had and those who had not had their blood pressure measured by sex (p = 0.213), age (p = 0.423), or SES index (p = 0.470).

### The Cascade of Care

The two phases of the study were completed by 370 (86.0%). There were significant differences between the group of those who completed the study and those who did not, according to sex and SES index (Table S1). Of those who completed the study, the majority were women (62.0%, p = 0.041) and from the middle (34.4%) and high (33.8%) SES index categories (p < 0.001). No differences were found between the two groups according to age (p = 0.423).

#### Step 1: Underwent the Medical Visit

**Figure 2:**
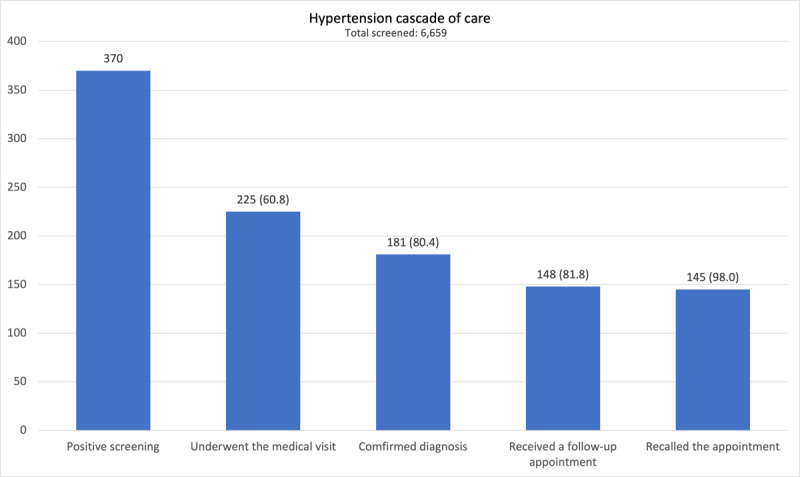
Number and percentage of patients meeting the inclusion criteria distinguished according to each step of the hypertension care cascade.

After the positive screening, 225 (60.8%) underwent the recommended medical examination (Figure 2). Reported barriers to accessing healthcare were lack of time (n = 89, 61.4%), lack of recommendations (n = 30, 20.7%), lack of symptoms (n = 17, 11.7%), lack of willingness (n = 8, 5.5%), and reported inability to locate the healthcare unit (n = 1, 0.7%).

**Figure 3:**
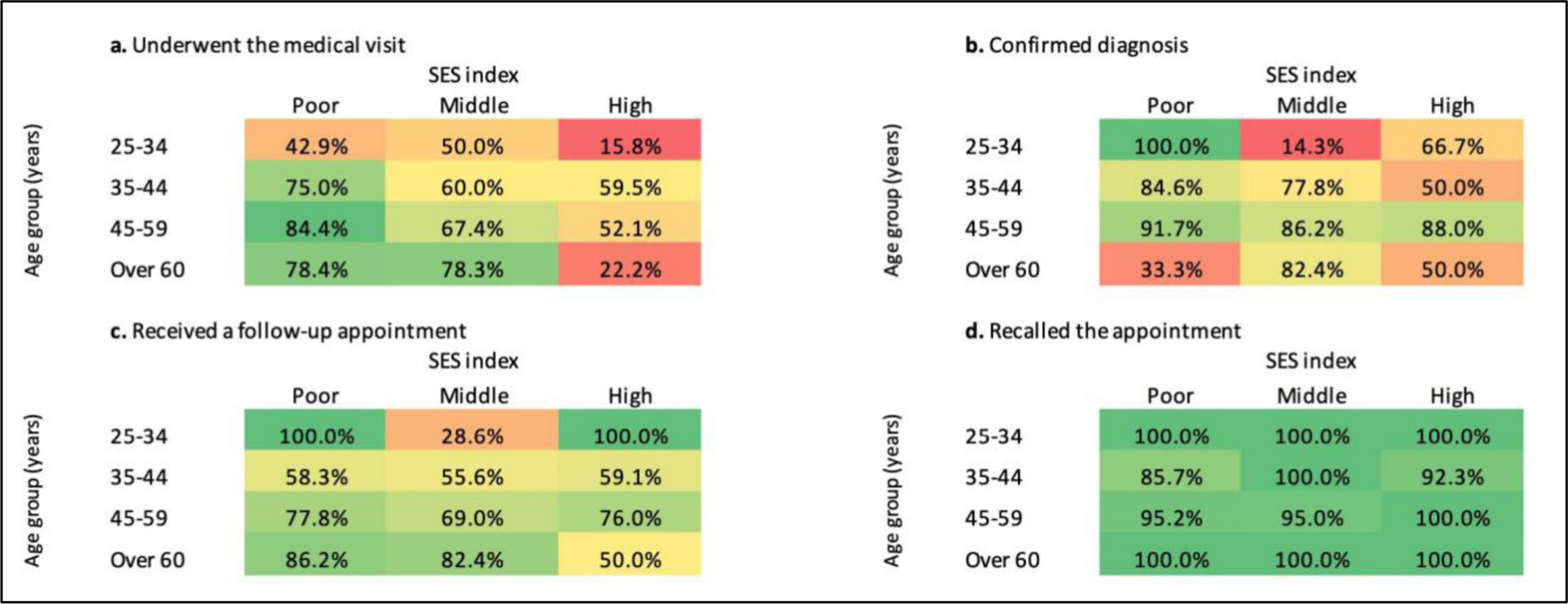
Proportion of participants with hypertension-positive screening who fulfilled each cascade step, stratified by age group, and SES index group. The color scale ranges from red (0.0%) to green (100%).

The probability of attending the visit was influenced by age and SES (Figure 3). Individuals aged over 35 years were more likely to attend the visit than those in the 25–34 age group (Table 2). The probability of attending the visit was lower for those from the high SES group compared to both middle (OR = 0.35, 0.95CI 0.16–0.76 p = 0.004 and low (OR = 0.21, 0.95CI 0.09–0.49, p < 0.001). No differences were found based on sex (p = 0.140).

**Table 2:** Results of the logistic regression models fitted on the four hypertension care cascade steps (yes/no) as dependent variables and age class, sex, and socioeconomic status (SES) index group as potential determinants. Post-hoc pairwise comparison was carried out through Tukey’s test. AG1 = 25–34 years, AG2 = 35–44 years, AG3 = 45–59 years, AG4 = over 60 years.


	OR	0.95CI	p-VALUE

**1 step – Underwent medical consultation**			

**Age groups (years)**			

AG2 – AG1	4.43	1.41–13.88	0.005

AG3 – AG1	3.95	1.41–11.03	0.003

AG4 – AG1	3.41	1.08–10.77	0.032

AG3 – AG2	0.89	0.38–2.11	0.986

AG4 – AG2	0.77	0.27–2.17	0.915

AG4 – AG3	0.86	0.35–2.11	0.974

**Sex(Female)**			

Male	1.56	0.87–2.23	0.140

**SES index**			

Middle – Low	0.59	0.26–1.32	0.270

High – Low	0.21	0.09–0.49	<0.001

High – Low	0.35	0.16–0.76	0.004

**2 step – Confirmed diagnosis**			

**Age groups (years)**			

AG2 – AG1	2.39	0.41–14.02	0.585

AG3 – AG1	9.34	1.61–54.24	0.006

AG4 – AG1	5.54	0.84–36.77	0.092

AG3 – AG2	3.91	1.08–14.15	0.033

AG4 – AG2	2.32	0.49–11.08	0.507

AG4 – AG3	0.59	0.13–0.74	0.816

**Sex(Female)**			

Male	1.37	0.58–3.39	0.487

**SES index**			

Middle – Low	0.28	0.08–1.02	0.054

High – Low	0.21	0.05–0.87	0.027

High – Low	0.75	0.23–2.43	0.831

**3 step – Received a follow-up appointment**			

**Age groups (years)**			

AG2 – AG1	0.73	0.13–4.01	0.964

AG3 – AG1	1.64	0.33–8.22	0.856

AG4 – AG1	2.81	0.47–16.90	0.449

AG3 – AG2	2.25	0.78–6.50	0.201

AG4 – AG2	3.84	0.98–15.03	0.055

AG4 – AG3	1.71	0.50–5.77	0.670

**Sex(Female)**			

Male	0.91	0.43–1.92	0.794

**SES index**			

Middle – Low	0.56	0.21–1.47	0.336

High – Low	0.92	0.30–2.80	0.981

High – Low	1.64	0.57–4.67	0.511

**4 step – Recalled the appointment**			

**Age groups (years)**			

AG2 – AG1	0.00	0–inf	1.000

AG3 – AG1	0.00	0–inf	1.000

AG4 – AG1	1.20	0–inf	1.000

AG3 – AG2	3.00	0.23–38.69	0.668

AG4 – AG2	1.09e^8^	0–inf	1.000

AG4 – AG3	3.64e^7^	0–inf	1.000

**Sex(Female)**			

Male	0.33	0.03–3.26	0.997

**SES index**			

Middle – Low	2.17	0.10–47.13	0.824

High – Low	4.31	0.18–100.68	0.522

High – Low	1.98	0.06–62.15	0.887


#### Step 2: Confirmed Diagnosis

A total of 181 out of 225 (80.4%) persons had the diagnosis of hypertension confirmed and 107 (59.1%) reported being informed about the diagnosis during the visit. The probability of having a hypertension diagnosis confirmed was influenced by age and SES, but not sex (p = 0.487).

The age group 45–59 years had a higher probability of a confirmed diagnosis than those aged between 25–45 years (Table 2). The probability of having the hypertension diagnoses confirmed was lower for both middle (OR = 0.28, IC95 0.08–1.02, p = 0.054) and high (OR = 0.21, 0.95CI 0.05–0.87, p = 0.027) SES groups compared to the low SES group.

#### Step 3: Received a Follow-up Appointment

Individuals who reported receiving a follow-up appointment were 148 (81.8%). This probability was not influenced by sex, age nor SES (Table 2).

#### Step 4: Recalled the Follow-up Appointment

Of the 148 patients who stated that they had a scheduled follow-up visit, only 3 (2.0%) could not remember the date. The next visits were scheduled after one month for 114 (78.6%) patients and the following week for 31 (21.4%). The probability of remembering the exact date of the follow-up was not influenced by sex, age, or SES group (Table 2).

## Discussion

### Main Findings

Through the analysis of the hypertension care cascade, this study evaluated the impact of a hypertension screening strategy (namely “hypertension corner”) implemented in two PHCCs in Quelimane, Mozambique. The first step (undergoing the recommended medical visit) was completed by 60.8% of individuals screened positive and 80.4% were confirmed with a diagnosis of hypertension (second step). The last two steps (receiving a follow-up appointment and recalling the appointment) were fulfilled by 81.8% and 98.0% of the patients in the previous step, respectively.

### Hypertension Burden and Screening Strategies in LMICs

Implementing new screening strategies for hypertension is crucial in LMICs, where chronic diseases are steadily increasing. In 2020, approximately 19.1 million deaths were attributed to CVD globally, with SSA being one of the regions with the highest mortality rates (345.8 to 475.7 per 100,000) [24,25]. Moreover, the African region is the WHO region with the highest prevalence of hypertension, at 27% [6]. In Mozambique, the prevalence of hypertension in young adults (aged 18–25) was 14.1% and 21.0% in women and men, respectively [8]. In the general population (aged over 20), a prevalence of 15.7% in women and 16.1% in men was observed [9].

The burden of hypertension on a vulnerable healthcare system is increased by the long-time hypertension can remain asymptomatic before presenting symptoms and complications. To decrease the burden of hypertension in these settings, it is essential to increase the rates of diagnosis, treatment, and disease control. The proposed targets for 2025 in Africa are to achieve a hypertension diagnosis rate of 80%, a treatment rate among diagnosed patients of 80%, and a disease control rate among treated patients of 80% [26]. The Hypertension Corner was effective in increasing the number of people the PHCC was able to include in the care cascade and in approaching the first two objectives mentioned above, as 60.8% of people with positive screening for hypertension went to the recommended visit and of these, 80.4% confirmed the diagnosis of hypertension.

By implementing different screening strategies, it is possible to reach wider segments of the population and increase awareness of hypertensive disease. A nationwide study conducted in Mozambique in 2015 found an awareness rate of hypertension of 14.5%, a treatment rate of 50.1%, and a control rate of 44.5% of treated patients [27]. Awareness in our sample was 38.5%; consisting of people who had been previously diagnosed with hypertension but were no longer under treatment at the time of the study. In addition, 34.9% of people had never had their blood pressure measured. Among the barriers to access to care reported in our study, those with the most striking support from the literature are the distance from health centers, lack of resources, and lack of adequate knowledge [28]. The implementation of awareness-raising interventions helps to increase treatment and control rates and to spread awareness of chronic diseases such as hypertension among the population [12].

### Hypertension Cascade of Care

In the first step of the care cascade, 60.8% of individuals with high blood pressure underwent the recommended medical examination. Previous studies in SSA have shown that population-based interventions for hypertension screening are an effective tool for improving mid-term outcomes in low-resource settings. [29]. The probability of undergoing the recommended visit was influenced by socioeconomic factors, with individuals with a lower SES being more likely to attend. This is an interesting finding, as higher SES is generally associated with greater access to care. Previous studies have shown that barriers to accessing screening for cervical and breast cancer in SSA include socioeconomic factors, such as low education, low income, and poor access to services [28,30]. The presence of a hypertension screening point at PHCC such as the HC could be a useful strategy to reach a disadvantaged population, i.e., those with low SES, who need tailored interventions to improve retention in care, as they are most at risk of not accessing health services.

Despite no differences being found based on sex, females are one of the populations most at risk regarding health provision and access. Indeed, in our sample, there were significant differences in SES between females and males the first having a lower level of education and lower-paying occupations. In sub-Saharan Africa, access to treatment is low for women, at 23% in East African states, resulting in females being disproportionately affected by CVD in terms of mortality, with a 15% excess of CVD deaths [31,32].

Individuals with a positive screening for hypertension were sent for a medical visit to the same PHCC where they were screened. Of the individuals who underwent the suggested examination, 80.4% were confirmed as having hypertension. The probability of diagnosis confirmation was lower for the “medium” and “high” SES categories than for the “low” category, and increased with age. Both age and socio-economic status are well-known risk factors in the literature [33]. Lower SES is associated with a higher risk of hypertension and higher blood pressure in LMICs, although some regional differences were found [34,35]. In particular, the greatest influence of SES on hypertension was due to the education level (pooled OR = 2.02, 0.95CI 1.55–2.63) and its burden was higher in women [36].

Once an individual enters the hypertension care cascade, it is crucial that they receive information about their condition and promote their retention in care. This is necessary to maintain high rates of adherence to therapy and, consequently, disease control. This approach contributes to reducing the long-term complications of the disease and its burden on one’s life and the health care system [37]. In our sample, 91.7% of patients with a confirmed diagnosis of hypertension received a follow-up appointment. This figure shows the possibility of healthcare providers to effectively attend to patients seen in the outpatient clinic and the PHCC’s capability to support these visits. In the last step of the care cascade, almost all those who had been scheduled for a follow-up visit remembered the visit date.

Access to care in Mozambique is also influenced by the geographical distribution of PHCC, as up to 66.7% of the population is underserved by these health services [38]. Therefore, investing in primary health care is key to ensuring universal health coverage. This can be done through continuous monitoring and evaluation of the PHC system [39].

### Study Limitations

The study has some limitations. First, the study was carried out in only two PHCCs in the city of Quelimane. Subsequent studies involving more facilities and different areas could strengthen the result and expand its generalizability Secondly, the group that completed the two phases of study differed from those lost to follow-up in terms of SES. This could result in selection bias. However, the three categories of SES identified were equally represented in the final sample. Lastly, data on follow-up visits and clinical outcomes were not collected so to better assess the effectiveness of this intervention future studies should include also these endpoints.

## Conclusions

Hypertension screening is critical in sub-Saharan Africa to address the increasing prevalence of hypertension, increase awareness and education about the disease, improve access to health services, and ultimately reduce the burden of cardiovascular diseases and their complications on individuals and societies. The hypertension corner, a screening tool for the health center-attending population, has proven to be a useful tool for effective hypertension screening with satisfactory retention in care, especially for people with lower socio-economic status. Nevertheless, given the low prevalence of hypertension found, future studies should conduct a cost-effectiveness analysis of this screening strategy. A screening point at the PHCC level can increase diagnosis and treatment rates and ultimately reduce the burden of non-communicable diseases on the health care system, reaching a vulnerable population at higher risk for NCDs and its complications.

## Data Accessibility Statement

The datasets generated and/or analyzed during the current study are available from the corresponding author on reasonable request.

## Additional File

The additional file for this article can be found as follows:

10.5334/gh.1339.s1 Annexs. Annex 1 and Annex 2.
