# Supplementary material for: Effectiveness of the Hypertension Screening Corner in Enhancing the Cascade of Care at Primary Healthcare Center Level: Evidence from Zambezia, Mozambique
Source: Glob Heart. 2024 Jul 10;19(1):58. doi: 10.5334/gh.1339 (PMC11243761; doi:10.5334/gh.1339)
Supplement: Annexs. — Annex 1 and Annex 2. [file gh-19-1-1339-s1.pdf]

ANNEX 1. The sociodemographic questionnaire

I. DEMOGRAPHICS
1.1. Sex: F □ M □
1.2. Age:
a. 25-34 □
b. 35-44 □
c. 45-59 □
d. 60 – 64 □
e. Over 64 years □

1.3 Education Level
a. No □
b. Primary □
c. Middle □
d. Secondary □
f. University or higher □

1.4 Marital status
a. Single □
b. Married □
c. De facto union □
d. Widow □
e. Separate □

1.5 Employment situation
a. Works in the public sector
b. Employment contract (salaried)
c. Self-employment (i.e., informal) □
d. Subsistence agriculture □
e. Work at home/ housewife □
f. Other: _____________

II. REASONS FOR THE VISIT TO THE HEALTH UNIT
2.1. What is the reason why you visit US?
a. Do you have a scheduled visit □
b. Accompanying/Caregiving: a family member □
c. Not feeling well □

2.2. If he/she had a consultation, please indicate where she had the visit:
a. HIV Consultation □
b. Pick-up Medications □
c. Other: ________________

2.3. If she/he is accompanying a family member, please indicate where you have the consultation:
a. Child Consultation □
b. HIV Consultation □
c. Pick-up Medications □
d. Other:____________________

2.4 If you are not feeling well, please indicate what symptoms you have:
the. Headache □
b. Dizziness □
c. Chest Pain □
d. Fever □
e. Coughs, and shortness of breath □
f. Other:_____________________

III. KNOWLEDGE ABOUT ARTERIAL HYPERTENSION
3.1. Have you heard on the radio or television about blood pressure or hypertension? YES □ NO □

3.2. Have you received a visit at your home from a community health workers or health professional who has told you about blood pressure or hypertension? YES □ NO □

3.3. What are the symptoms of a person with high blood pressure? (multiple choice)
a. Headache □
b. Dizziness □
c. Has no symptoms □
d. Don't know □
e. Other: __________

3.4 Is it important to use low quantities of salt in food??
a. Very important □
b. Important □
c. Not important □
d. I don't know □

3.5. During your work, do you practice physical activity (walking for more than an hour, lifting heavy objects, fishing, farming)? YES □ NO □

3.6. Currently you smoke any type of tobacco (i.e., cigarette)
YES □ NOT □

3.7. Have you consumed any alcoholic drinks in the last week?
YES □ NO □

3.8. Do you carry out any sports activities?
YES □ NO □
If YES indicate the frequency per week: _________

IV. HISTORY OF ARTERIAL HYPERTENSION
4.1. Have I done any other blood pressure control?
YES □ NO □

4.2. Have you been told by a health professional in the last year that you suffer from hypertension? YES □ NO □

4.3. Do you have any family members with hypertension?
YES □ NO □

2.3.1. If YES Specify the type of relationship level _________

4.4. Have you ever taken any antihypertensive drugs? YES □ NO □
4.4.1 If YES, where you usually get treatment or counselling for the high blood pressure (multiple response)
a. Health Center □
b. Provincial Hospital □
c. Private hospital □
d. Private pharmacy □
e. Other: _______________

4.5. Have you visited a traditional doctor for your blood pressure? YES □ NO □

4.6 Are you currently taking traditional medicine for high blood pressure? YES □ NO □

V. SOCIO-ECONOMIC ASSESSMENT OF THE FAMILY

5.1. How many people live in the house? (Write in numbers, counting the parents and including the respondent)
N Total people ____________
N° children under 14 years _______
N° personas over 14 years old _______

5.2. Does the house have a perishable roof?
YES □ NO □

5.3. The house has floor with washable surface (cement, wood, tiles, others) YES □ NOT □

5.4. Is there a radio at home? YES □ NO □

5.5. Are there any means of transport at home?
YES □ NO □

5.5.1. If YES, what kind? (put "X" in the answers, multiple choice)
a. Bicycle □
b. Motorbike □
c. Car □
d. Other: ________________

5.6. Is there a source of energy/light at home? YES □ NO
5.6.1. If YES, which one?
a. Electric current □
b. Electric generator □
c. Oil lamp □
d. Battery-operated flashlight □
e. Other: ___________

5.7. Where does most of the family's income come from?
a. Subsistence agriculture □
b. Agriculture for commercial purposes □
c. Informal work □
d. Employment contract (salaried) □
e. Poultry breeding / fishing □
f. Another: ____________

5.8. What is the main source of water?
a. Running water at home □
b. Public water points (well, fountain) □
c. Natural source (rain, river, lake) □
d. Other: _____________

5.9. What kind of sanitary facilities does the house have?
a. None □
b. Of perishable material (earth, branches, wood, hole in the ground) □
c. With washable floor (latrine) □
d. With drain and septic tank □

ANNEX 2. Questionnaire on the management and treatment of hypertension

I. CLINICAL EVALUATION PERFORMED ON THE SAME DAY OF THE SCREENING (In case of an interview made on the same day that passed through the corner)

6.1. Have any antihypertensive drugs been prescribed? YES □ NO □
6.1.1. If YES, can you specify which ones?
A________________________
B_________________________
C_________________________

6.2. Have you received a user card? YES □ NO □

6.3. Have you received information about when to return to visit? YES □ NO □

6.4. Have you been informed about the disease you have been diagnosed with? YES □ NO □

6.5 When will you get to go back to the health unit?
a. Next week □
b. From here to a month □
c. When you feel sick □
d. Don't know □
e. Did not answer □

6.6. Do you use any means of transport to go to the health unit? YES □ NO □

6.6.1 If YES, what means of transport do you use to get to the health unit?
a. Bicycle □
b. Motorbike □
c. Car □
d. Bus/public transport □
e. Other: ________________

6.7 How long does it take to go to the health unit?
a. Less than an hour □
b. 1 to 2 hours □
c. 3 hours or more □

II. EVALUATION AFTER TWO WEEKS (Did not attend the clinical consultation of chronic disease, In case of interview made after 2 weeks of the day that passed through the corner)

7.1. The last time you were at US after blood pressure was measured in the Hypertensive Corner, was it evaluated again by any clinician? YES □ NO □

If YES, it follows 7.2 and others (minus 7.9), if NO, it goes to 7.9 and follows until the end

7.2. If YES, where was it assessed?
a. at the chronic disease's consultation □
b. I don't remember □
c. In another setting. Specify which: _______

7.3. Have any antihypertensive drugs been prescribed? YES □ NO □
7.3.1. If YES, can you specify which ones?
A________________________
B_________________________
C_________________________

7.4. Have you received a user card? YES □ NO □

7.5. Have you received an indication of when to return? YES□ NOT □

7.6. Are you taking antihypertensive drugs? YES □ NO □

7.7. Have you been informed about the disease you have been diagnosed with? YES □ NO □

7.8. When will you get to go back to the health unit?
a. Next week □
b. From here to a month □
c. When you feel sick □
d. Don't know □
e. Did not answer □

7.9. If NOT, explain why you did not go to the medical visit?
a. No one remembered me to go to the medical visit □
b. In the Office of External Consultation there were many people waiting, and I gave up □
c. Did not find the location of the consultation □
d. I went to the visits, but the clinician was not □
e. I didn't feel like going □
f. I had another appointment / accompany the family member □
g. I didn't have time □

7.10. Do you use any means of transport to go to the health unit? YES □ NO □

7.10.1. If YES, which means of transport do you use to get to the health unit?
a. Bicycle □
b. Motorbike □
c. Car □
d. Bus/public transport □
e. Other: _______________

7.11. How long does it take to go to the health unit?
a. Less than an hour □
b. 1 to 2 hours □
c. 3 hours or more □
